# Perceived Stress, Knowledge, and Preventive Behaviors in Indian versus US-based Participants During COVID-19: A Survey Study

**DOI:** 10.3389/fpubh.2021.687864

**Published:** 2021-09-13

**Authors:** Ankita Sinharoy, Shekhar Pal, Jishu Das, Pritish Mondal

**Affiliations:** ^1^School of Business Administration, Penn State University, Harrisburg, PA, United States; ^2^Department of Tropical Medicine, School of Tropical Medicine, Kolkata, India; ^3^Department of Internal Medicine, The Ohio State University, Columbus, OH, United States; ^4^Department of Pediatrics, Division of Pediatric Pulmonology, Penn State College of Medicine, Hershey, PA, United States

**Keywords:** COVID-19, COVID-related perception, hydroxychloroquine, perceived threat, comparative study, COVID-related stress, COVID-19 knowledge, COVID preventive behavior

## Abstract

**Rationale:** India and the USA, the worst affected countries by COVID-19, experienced very different pandemic courses. By 2020, COVID-19 cases had steadily declined in India, whereas the fight continued in the US. The people of India and the USA perhaps perceived threats very differently, influenced by their knowledge, available healthcare facilities, and social security. We conducted an online survey study to compare COVID-related perceptions between Indian participants (IND-P) and US-based participants (US-P).

**Methods:** COVID-related perceptions such as stress, knowledge, and preventive behaviors were measured with specific questionnaires, and normalized scores were computed. *T*-tests were used to compare the perception scores, while the Kruskal-Wallis-H (KWH) tests were used to compare socioeconomic distributions between participants from two countries. Generalized linear model (GLM) adjusted for sociodemographic confounders estimated the association between the country of residence and COVID-perception.

**Results:** The IND-P (*N* = 242) were younger and male-dominated compared with the US-P (*N* = 531) (age: KWH = 97.37, *p* < 0.0001, gender: KWH = 140.38, *p* < 0.0001). Positive attitudes toward preventive guidelines were associated with higher perceived risk and stress (*r* = 0.35, *p* < 0.001, and *r* = 0.21, *p* < 0.001, respectively) but not with the knowledge (*r* = −0.05, *p* = 0.14). Compared with the US-P, the IND-P had lower knowledge (5.19 ± 1.95 vs. 7.82 ± 1.35; *t*-test: p < 0.0001), higher stress (7.01 ± 1.51 vs. 6.07 ± 1.61; *t*-test: *p* < 0.0001), and better adherence to preventive guidelines (8.84 ± 1.30 vs. 8.34 ± 2.09; *t*-test: *p* = 0.0006). GLM demonstrated a significant association between the country and COVID-perception scores.

**Conclusion:** The IND-P experienced higher stress and perceived threat during COVID-19 than the US-P, perhaps due to a lack of faith in the healthcare system and insecurity. Despite lower knowledge, the IND-P had better acceptance of preventive guidelines than the US-P.

## Introduction

Daily census and mortality count are suboptimal representations of the devastation of COVID-19 (1). While living under the darkness of the pandemic, the lives of the millions have changed forever. Even the people who did not have the disease suffered from the threat of contracting COVID-19, financial stress, grief, and depression. India and the USA, two of the largest populous nations, were affected most by the pandemic. However, the confirmed cases and mortality due to the COVID-19 were double in the USA compared with India till the end of 2020 (2). Toward late 2020, COVID-19 cases had steadily declined in India, although the battle continued in the USA (2). While the factors attributing to the different courses are unknown, perceptions and behavior of people toward COVID-19 may hold some answers.

The administrative support, social structures, available healthcare facilities, and family income contrast India and the USA, as reflected by the human development index ranking of 131 and 17, respectively (3). Thus, people of these two countries perhaps experienced COVID-related stress and threat differently. Since COVID-19 was not confined to political and geographic boundaries, population-based research beyond the regional level would help introspect the differences and inform unique strategies based on the diverse necessities.

The healthcare expenditure in the USA is the highest globally, and the USA could afford to maximize COVID-19 testing and upgrade healthcare facilities to match the demand of the pandemic (4). India too conducted millions of COVID-19 testing, and the numbers were only second to the USA (5). However, the Indian healthcare system was overwhelmed during the peak of COVID-19 (5). In contrast, suboptimal healthcare infrastructure was already a stressor in India; severe economic recession, unemployment, home quarantine, and inadequate administrative support had worsened the situation further (6). In this scenario, COVID-related information from authentic sources and awareness could have helped mitigate some apprehensions (7). While websites like CDC (Centers for Disease Control and Prevention) played a major role in spreading awareness in the USA, Indians often relied on social media to learn about COVID-19. In March 2020, social media activities grew by 50 times in India (8). Some of the Indian news channels played a negative role by an upsurge of COVID-19 related information and misinformation, which worsened the preexisting fear and uncertainties about the disease outcome (9, 10).

Preventive measures have been the best way to curb the spread of COVID-19, especially when effective therapy or vaccine was unavailable. Even after the arrival of the COVID-19 vaccine, it is expected to take a considerable amount of time to develop adequate herd immunity (11). The “perceived threat” of an individual has been reported as a major determinant for adherence to the preventive measures, as reported during the 2009 swine flu pandemic, and also during COVID-19 (12, 13). Based on diverse socioeconomic conditions in India and the USA, people in these two countries probably had differences in perceived threats of COVID-19, which has also been considered a determinant of COVID-vaccine acceptance (14, 15).

Several novel therapies for COVID-19 had been promoted worldwide, such as hydroxychloroquine, diethylcarbamazine, azithromycin, and herbal medicine, without any proven benefit (16). Hydroxychloroquine had received major public attention in India and the USA (17, 18). While Indians favored hydroxychloroquine since the Indian Council of Medical Research, the highest medical governing body had approved its use (19), public opinion was deeply divided in the USA during the early days of the pandemic. The public perception of the effectiveness of hydroxychloroquine had never been compared between India and the USA, an interesting question to be asked to the community.

We conducted a survey study with the primary objective of comparing COVID-related perceptions such as stress, knowledge, and preventive behaviors between the Indian participants (IND-P) and the US-based participants (US-P). The secondary objective was to compare perceived threats and attitudes toward COVID-vaccine and hydroxychloroquine between the two groups. We hypothesized that higher COVID-related stress and perceived threat would positively correlate with a better attitude toward preventive recommendations among survey participants. We further hypothesized that the IND-P compared with the US-P would have higher COVID-related stress, perceived threat, and a better attitude to preventive recommendations, including COVID-vaccine and hydroxychloroquine.

## Materials and Methods

### Redcap Survey

We designed a Redcap survey to test COVID-related perceptions, namely, stress, knowledge, and attitude to the preventive recommendations. Stress-related questions were based on confinement, job loss, availability of healthcare facility, contracting COVID-19, and risk of death, whereas the knowledge-related questionnaire was focused on understanding disease transmission, prevention, and treatment. We evaluated the attitudes of people toward preventive guidelines such as wearing face-mask, social distancing, handwashing, and lockdown. We built the survey questionaries (Supplemental File 1) incorporating the key elements of the health belief model (HBM), a well-recognized model that helps to quantify the risk perception and specific behavior of people toward health-related conditions (20). HBM is constructed based on various perceptions about the outcome of a disease condition, namely, susceptibility, severity, benefits of action, barriers, self-efficacy, cues to action, and preventive behaviors (21, 22). The specific questions are displayed in Supplemental File 2 (stress-related questions: 13–19), Supplemental File 3 (knowledge-related questions: 31–58), and Supplemental File 4 (questions on preventive behavior: 20–24). The stress and preventive practice questions were primarily framed on the Likert scale (1–5), whereas knowledge-related questions were mostly dichotomous (yes vs. no). We also added questions on satisfaction with healthcare facilities, including hospitals, ventilator availability, and administrative initiative. Participants aged below 18 years, living outside of India or the USA, or of unknown residence were excluded from the study analysis. Penn State College of Medicine Institutional Review Board approved the study protocol.

### The Enrollment of The Survey Participants

Researchmatch, an online portal, was the primary mode of requirement in the US (23). We randomly sent Redcap survey requests to a pool of 1,50,000 volunteers across all 50 US states, available in Researchmatch. However, a similar web-based tool was not available in India. Thus, we primarily relied upon social media to recruit the IND-P and distributed the survey among several Facebook groups, which belonged to 44 different Indian cities, spread out across the country. Although this was an anonymous survey, we received several post participation feedback, which helped us recognize the diverse geographic distribution of the IND-P. However, per study protocol, we did not collect personal identifiers such as city or zip/pin code. All the participants electronically signed informed consent. The responses were collected between the end of May 2020 and early October 2020.

### Sociodemographic Groups

Study respondents were categorized based on various socioeconomic factors, such as (Table 1): age (group I to V, in ascending order), gender, education level (five groups: high school or below, undergraduate, graduate, masters, and PhD/MD), and family income (four groups). We further queried whether the participant was a healthcare worker and willing to accept the COVID-19 vaccine or not. Finally, we inquired about the resources used by the participants to learn about COVID-19, such as social media, television, official health websites like WHO, CDC, and personal communications.

**Table 1:** The distribution of sociodemographic factors and comparison between Indian and US-based participants using Kruskal-Wallis Test.

**Sociodemographic factors**		**Country**		**Kruskal-Wallis Test**		
		**India**	**USA**	**df**	**Kruskal-Wallis H**	***P*-value**
Age	Group I (18-24 years)	52 (21.6%)	40 (7.6%)	4	97.37	<0.0001
	Group II (25-44 years)	155 (64.3%)	221 (41.9%)			
	Group III (45-60 years)	25 (10.4%)	171 (32.4%)			
	Group IV (61-70 years)	6 (2.5%)	63 (12.0%)			
	Group V (>70 years)	3 (1.2%)	32 (6.1%)			
Education Level	High School	6 (2.5%)	76 (14.5%)	4	35.28	<0.0001
	Undergraduate	28 (11.7%)	143 (27.3%)			
	Graduate	76 (31.8%)	103 (19.7%)			
	Masters	81 (33.9%)	119 (22.7%)			
	PhD./ Professionals	48 (20.1%)	83 (15.8%)			
Family Income	Lower-middle class	24 9.9%)	51 (9.7%)	3	2.05	0.153
	Middle class	157 (64.9%)	313 (59.4%)			
	Upper-middle class	59 (24.4%)	153 (29.0%)			
	Rich	2 (0.8%)	10 (1.9%)			
Gender	Male	148 (61.7%)	97 (18.6%)	1	140.38	<0.0001
	Female	92 (38.3%)	425 (81.4%)			

*df, degree of freedom; p < 0.05 is considered significant*.

### Computing Perceived Threat and Dimension Reduction

The data was analyzed using SPSS 27 and SAS 9.4 (24, 25) and shared at Mendeley (26). A new variable was computed based on the determinants that could affect the perceived risk of an individual on COVID-19. The factors used to compute “perceived risk” included age (60 years or above), healthcare worker (yes vs. no), family member diagnosed with COVID-19 worker (yes vs. no), access to adequate healthcare, COVID-19 status in the state of residence, and the likelihood of having the severe disease if contracted the virus. We used the “dimension reduction” function in SPSS (27) to abbreviate the metrics on stress, knowledge, perceived risk, and preventive guideline adherence into four normalized scores (continuous variable) with a range between “0 and 10”.

### Time Trend Analyses

Since this survey was conducted over 6 months, COVID-related perceptions could have changed dynamically over that period of time. Thus, we conducted time trend analyses for knowledge, stress, and preventive behavior in both the groups, using a time-series modeler (28). We conducted the Ljung-Box Q test to test the null hypothesis “COVID-related perceptions do not have auto-correlation with the study period”.

### Comparative Analyses

The primary outcome of this study was the differences in knowledge, stress, preventive behaviors, and perceived threat between the IND-P and the US-P. Chi-square test was used to compare dichotomously distributed socioeconomic factors like gender, whereas the Kruskal–Wallis test was used to compare the ordinal variables, such as age, family income, education level, and hydroxychloroquine effectiveness between two groups. The Non-parametric Kruskal–Wallis test was used since it was the test of choice when ordinal variables were compared between two independent groups (IND-P vs. US-P) (29). We used two-sample *t*-tests to compare the continuous variables such as stress, knowledge, and preventive behavior scores between the participants of the two groups.

### Generalized Linear Model and Cross-Validation

As the next step of data analysis, the association between country of residence (India vs. the USA) and COVID-related perception was estimated using a generalized linear model (GLM). Since the demographic distributions were very different between the two groups, GLM was adjusted for confounders such as education level, gender, family income, and age. GLM was built on randomly selected 70% of the study participants (training group), whereas the remaining 30% subjects (testing group) were used to validate the model. We used GLM instead of simple linear regression since some of the independent variables such as gender and country of residence were categorically distributed.

## Results

### Socioeconomic and Demographic Distribution

Redcap captured 962 responses, and 189 respondents were excluded since their country of residence was neither India nor the USA or unknown. Of the remaining 773 valid responses, 242 and 531 participants were from India and the USA, respectively. The majority of the US-P (41.9%) were in age-group II (24–44 years) and age-group III (45–60 years) (32.4%), whereas among the IND-P, 64.3% were in age-group II, followed by age-group I (18–24 years) (21.58%) (Table 1). The age-wise distribution of participants was statistically different between the two groups, as the average IND-P were younger compared with the US-P (Kruskal–Wallis Test: KWH: 97.37, *p* < 0.001). The IND-P were predominantly male (61.7%), in contrast to a higher number of female respondents (81.4%) among the US-P, and the gender distribution was significantly different between two groups (Pearson's chi-square = 204.94, *p* < 0.0001) (Table 1). We did not find a difference in family income between the IND-P and the US-P (KWH: 2.05, *p* = 0.153), as the middle-class followed by the upper-middle-class were predominant in both the groups (59.4 and 29.0% in the USA vs. 64.88 and 24.38% in India) (Table 1). The IND-P and the US-P also had a significant difference in education level (KWH: 35.28, *p* < 0.0001). Among the IND-P, 85.8% reported having a bachelor degree or above, compared with 58.2% among the US-P (Table 1). The US-P were predominantly whites (80.4%), and the remaining participant pool was comprised of seven other races, whereas all the IND-P but one was Asian-Indian.

### Unmatched Sample Size and Power Analysis

Considering an unmatched sample size between the IND-P and the US-P, a power analysis was conducted on two-sided independent sample *t*-tests that compared each of the COVID-perception metrics (stress, knowledge, and preventive behavior) between the two groups. With an α = 0.05, the projected power for all three tests was well above the significance cut-off of 0.8 (Supplemental Table 1).

### Time Trend Analyses

COVID-related perceptions did not change significantly over time (study period) in both the study groups. The degree of associations between the study period and COVID-related perceptions (knowledge, stress, and preventive behavior) was not statistically significant in the IND-P (0.978, 0.564, and 0.300, respectively) or the US-P (0.126, 0.722, and 0.123, respectively).

### Preventive Behaviors

**Figure 1:**
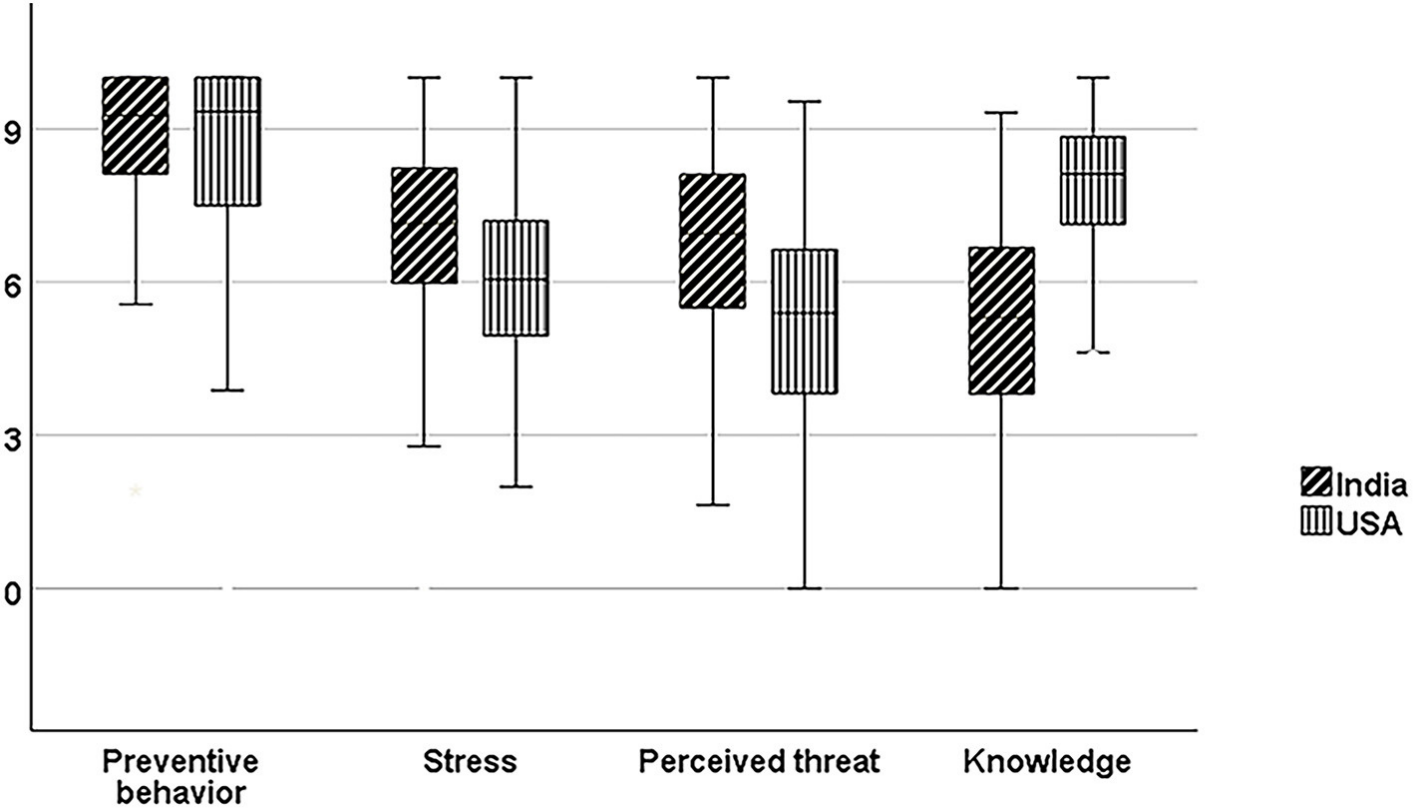
Boxplots with error bars representing comparative scores of preventive behaviors, stress, perceived threat, and knowledge between the study participants of India and the USA. The scores are normalized on a “0–10 scale”, and the horizontal lines within the box represent the median value.

Positive attitudes toward preventive behaviors were associated with higher perceived risk and stress (*r* = 0.35, *p* < 0.001, and *r* = 0.21, *p* < 0.001, respectively) but not with the knowledge (*r* = −0.05, *p* = 0.14). The IND-P achieved a lower knowledge score (5.19 ± 1.95) compared with the US-P (7.82 ± 1.35), based on a *t*-test (*p* < 0.0001) (Figure 1). In contrast, the IND-P reported higher stress (7.01 ± 1.51) compared with the US-P (6.07 ± 1.61) (*t*-test: *p* < 0.0001) (Figure 1). Stress scores were also higher in women (6.18 ± 1.64) compared with men (5.65 ± 1.50) among the US-P (*t*-test: *p* = 0.0035) but not among the IND-P (*p* = 0.10). The IND-P (8.84 ± 1.30) demonstrated a better attitude toward preventive guidelines vs. the US-P (8.34 ± 2.09), and the difference was statistically significant (*t*-test: *p* = 0.0006) (Figure 1). The IND-P (6.72 ± 1.78) also reported a higher perceived threat than the US-P (5.20 ± 2.04) (*t*-test: *p* < 0.0001) (Figure 1). The IND-P identified television (79.34%) as their preferred source of information. However, other notable options like the WHO website (45.45%) and city/state websites (35.54%) were less popular among the IND-P. Television was the most popular source of information among the US-P (65.91%) too, closely followed by city/state websites (65.54%) and CDC websites (64.60%).

### GLM and Cross-Validation

Training cohort: GLM demonstrated that the country of residence had significant associations with preventive behaviors, stress, and knowledge scores (*p* = 0.005, *p* < 0.001, *p* < 0.001, respectively), whereas it was adjusted for age, gender, education level, and family income (Table 2).

**Table 2:** Generalized linear model (GLM) demonstrating a significant association between country of residence of the participants (India vs. the USA) and COVID-related perceptions, while adjusted for gender, education level, family income, and age.

**Dependent variables (COVID-related Perceptions)**	**Independent variables**	**Training sample**				**Testing sample**			
		**Beta**	**95% Confidence Interval**	**Wald Chi-Square**	**Sig**.	**Beta**	**95% Confidence interval**	**Wald Chi-Square**	**Sig**.
Knowledge	Country	−2.618	−2.982, −2.254	198.827	<0.001	−2.648	−3.146, −2.149	108.365	<0.001
	Gender	0.175	−0.160, 0.510	1.052	0.305	0.040	−0.440, 0.520	0.027	0.870
	Education level	0.120	−0.004, 0.244	3.589	0.058	0.048	−0.112, 0.208	0.346	0.557
	Family income	0.321	0.083, 0.559	6.995	0.008	0.458	0.117, 0.798	6.930	0.008
	Age	−0.028	−0.183, 0.128	0.121	0.728	0.002	−0.210, 0.213	0.000	0.988
Stress	Country	0.756	0.414, 1.099	18.754	<0.001	1.184	0.673, 1.694	20.620	<0.001
	Gender	0.197	−0.123, 0.517	1.460	0.227	0.250	−0.222, 0.723	1.079	0.299
	Education level	0.054	−0.062, 0.171	0.827	0.363	0.012	−0.156, 0.179	0.018	0.893
	Family income	−0.342	−0.567, −0.117	8.859	0.003	−0.437	−0.772, −0.102	6.536	0.011
	Age	−0.111	−0.258, 0.035	2.220	0.136	−0.096	−0.316, 0.125	0.722	0.396
Preventive behavior	Country	0.591	0.183, 1.000	8.042	0.005	0.810	0.220, 1.400	7.248	0.007
	Gender	0.454	0.065, 0.843	5.228	0.022	0.674	0.151, 1.197	6.391	0.011
	Education level	0.098	−0.041, 0.237	1.918	0.166	0.202	0.006, 0.397	4.073	0.044
	Family income	0.230	−0.044,0.503	2.705	0.100	0.079	−0.297, 0.455	0.169	0.681
	Age	−0.017	−0.189, 0.154	0.040	0.841	0.189	−0.075, 0.453	1.964	0.161

Testing cohort: The adjusted model was cross-validated with a testing group. The degree of association between country of residence with preventive behavior, stress, and knowledge (*p* = 0.007, *p* < 0.001, *p* < 0.001, respectively) were very similar to the training group (Table 2).

### Estimation of Other Concerns and Behaviors

The IND-P, compared with the US-P, consistently reported higher concerns about inadequate healthcare facilities, including hospitals, ventilator availability, and administrative initiative (based on Kruskal–Wallis Test) (Table 3). A significantly higher number of IND-P than the US-P believed that hydroxychloroquine might prevent the disease and were ready to participate in future drug or vaccine trials (Table 3). The attitudes of the participant toward the COVID-19 vaccine were also varied, as 94.12% of the IND-P were willing to accept COVID-19 immunizations compared with 78.81% of the US-P (Pearson's chi-square = 27.93, *p* < 0.0001). In both groups, participants willing to take the COVID-19 vaccine reported a higher perceived threat than those who had declined.

**Table 3:** Comparative analyses of perception scores about the local healthcare facilities, hydroxychloroquine effectiveness, and other preventive behaviors between Indian and US-based participants (US-P) during COVID-19.

**Concern**	**India**		**USA**		**Kruskal-Wallis Test**		
	**Mean rank**	***N***	**Mean rank**	***N***	**Kruskal-Wallis H**	**Df**	***p*-value**
Hydroxychloroquine effectiveness	518.29	238	320.21	525	140.256	1	<0.0001
Drug trial participation	406.36	238	369.46	523	4.853	1	0.028
Vaccine trial participation	409.09	238	370.47	526	5.271	1	0.022
Available healthcare	223.72	238	456.70	530	198.029	1	<0.0001
Ventilator support availability	193.28	239	469.77	527	269.219	1	<0.0001
Support from administration	351.92	239	397.82	527	7.459	1	0.006

*The Kruskal–Wallis test was used for the analyses. Indian participants (IND-P) had higher faith in hydroxychloroquine, were more willing to participate in drug or vaccine trials, and were less satisfied with the available healthcare system than the US-P*.

## Discussion

The difference in the sociodemographic characteristics between the IND-P and the US-P was one of the features of this internet-based survey. The majority of the IND-P belonged to younger age groups. This trend perhaps reflected the ever-growing popularity of the internet among the tech-savvy younger Indian generation, whereas the older Indian population was probably reluctant to learn new technologies. In contrast, the entire US population had access and was comfortable with online portals, and thus, the US respondents maintained a relatively uniform age-wise distribution compared with the IND-P.

The US-P were predominantly women, and the gender difference followed a similar trend described in prior surveys where women dominated the survey participation (30, 31). A recent global study found that women were three times more likely than men to suffer from serious mental health issues during this pandemic, and amplified COVID-related stress perhaps encouraged higher female participation in the US (27). However, a similar trend was not seen in India, which could be due to male predominance in internet access and usage (32).

This study demonstrated a higher stress level among the IND-P compared with the US-P. In 2020, India had witnessed the worst recession in recent times (33). COVID-related stress was augmented by several factors, including job loss, pay cuts, lack of adequate healthcare facilities, and confinement. Moreover, preexisting poverty, unemployment, and lack of social security altogether resulted in an exponential increase in job-related stresses and insecurities to fulfill basic needs (9). Additionally, a continuous upsurge of information from social media, news channels, and mobile notifications, with an overburdened and inadequate healthcare system, possibly heightened the fear more among the IND-P than the US-P (9).

The US-P demonstrated a better COVID-related knowledge than the IND-P, which could be related to greater awareness and better access to authentic information in the USA. Although most of the IND-P indicated that television was their preferred source of information, the quality and authenticity of the information provided could greatly vary among the countries. On the contrary, many of the US-P chose official state websites and CDC websites, which offered correct information reflected in their knowledge scores.

The IND-P, compared with the US-P, reported a higher perceived threat, had a better attitude to the preventive guidelines, and were more willing to accept the COVID-19 vaccine or participate in future drug or vaccine trials. The perceived threat has been identified as a key determinant of compliance with preventive guidelines in previous COVID-19 studies (34). We found a similar association between the individual perceived threat and positive attitude toward the preventive guidelines, which further established that observation. Finally, higher faith in hydroxychloroquine among the IND-P could be influenced by the administrative approval in India and desperation to prevent COVID-19 infection.

We conducted this comparative study before the COVID-vaccine was launched in late 2020, and a new wave of pandemics hit the USA around the same time (35, 36). Our study demonstrated that the IND-P were more willing to accept COVID-vaccine compared with the US-P. Interestingly, COVID-vaccine hesitancy was later recognized as the foremost public health challenge in the USA (37), whereas many Indians could not be vaccinated, despite willingness, because of inadequate availability (38). In both countries, new waves of COVID-19 were facilitated due to the relaxation of preventive behaviors. India enjoyed initial success in containing COVID-19, prematurely declared a victory, followed by a preventive behavior collapse in early 2021 (39). Subsequently, India, without adequate immunization coverage, faced the worst global COVID-19 crisis in April 2021 due to the delta variant of SARS-CoV-2 (40). The concern with lack of healthcare facilities, expressed by the IND-P of our survey, was proven justified, as India struggled to provide even basic medical facilities to the staggering numbers of morbid patients with COVID-19 (41), and the death toll was sky-high between April and June 2021 (42). While in the USA, a significant percentage of the population were confident with available healthcare and were reluctant to accept the COVID-19 vaccine. Now they became vulnerable to the delta variant of SARS-CoV-2, as the country is currently facing (August 2021) resurgence of COVID-19 cases due to the delta variant (43).

One of the limitations of this study is generalizability. Considering the diverse and vast population of India and the USA and the moderate sample size of this survey, the results may not represent the general population. Instead, this study report should be considered as a trend and needs to be externally validated with a large sample survey prior to accepting the results as a general trend. Nonetheless, we have made considerable efforts to reach out to people across India and the USA. Since the difference in sociodemographic distribution between the IND-P and the US-P was significant, we adjusted GLM for potential sociodemographic confounders. However, building a prediction model was not the goal of this study. Finally, the study questions were in English, and about 90% of Indians were not comfortable with that language (44). Since the study was conducted during the pre-vaccine period, we could not capture the change in COVID-related perceptions during the post-vaccination period, including vaccine hesitancy in the USA. Moreover, the survey was conducted in 2020 and did not capture the devastating impact of the delta variant in India.

Nonetheless, this study has few strengths. This survey is among few epidemiological studies that compared people's perception of COVID-19 between India and the USA, representing the developing and developed worlds. This study demonstrated an association among preventive behaviors, stress, and perceived threat, and despite lower knowledge, the IND-P had a better attitude toward preventive guidelines than the US-P, which could be related to higher stress. However, it is unknown whether better adherence to preventive guidelines helped India achieve better initial success against COVID-19 than the USA. Finally, enthusiasm to participate in future drug or vaccine trials among the IND-P has conveyed an important message to the regulatory bodies to consider.

## Conclusion

The IND-P perceived COVID-19 as a higher threat than the US-P, perhaps due to a lack of faith in the available healthcare system and social security. Driven by stress and perceived risk during the pandemic, IND-P possibly were more inclined to accept the preventive measures, including COVID-vaccine and unproven therapies like hydroxychloroquine, than the US-P. The causal association between the preventive practice and the pandemic course was beyond the scope of this study. A nationwide survey with a larger sample size in the future is necessary to understand whether better compliance to the preventive guidelines had helped India with initial success against COVID-19 compared to the USA, in 2020.

## Data Availability Statement

The datasets presented in this study can be found in online repositories. The names of the repository/repositories and accession number(s) can be found below: https://data.mendeley.com/datasets/hp3pdpjgrs/2.

## Ethics Statement

The studies involving human participants were reviewed and approved by Penn State College approved the study of Medicine Institutional Review Board (approval ID: STUDY00015136), and all methods were performed in accordance with the relevant guidelines and regulations. The patients/participants provided their written informed consent to participate in this study.

## Author Contributions

AS, SP, JD, and PM: conceptualization, writing—review, editing, validation, methodology, and investigation. AS and PM: visualization, data curation, formal analysis, software, and writing. JD and PM: supervision. AS: project administration. PM: funding acquisition and resources. All authors contributed to the article and approved the submitted version.

## Conflict of Interest

The authors declare that the research was conducted in the absence of any commercial or financial relationships that could be construed as a potential conflict of interest.

## Publisher's Note

All claims expressed in this article are solely those of the authors and do not necessarily represent those of their affiliated organizations, or those of the publisher, the editors and the reviewers. Any product that may be evaluated in this article, or claim that may be made by its manufacturer, is not guaranteed or endorsed by the publisher.

## Abbreviations

HCW: Healthcare worker
df: degree of freedom
IND-P: Indian participants
US-P: US-based participants.
