# Supplementary material for: Perceived Stress, Knowledge, and Preventive Behaviors in Indian versus US-based Participants During COVID-19: A Survey Study
Source: Front Public Health. 2021 Sep 13;9:687864. doi: 10.3389/fpubh.2021.687864 (PMC8473728; doi:10.3389/fpubh.2021.687864)
Supplement: Supplementary file 1 [file Data_Sheet_1.PDF]

COVID-19 SURVEY

PID 7859

Codebook

Data Dictionary Codebook

03/25/2021 4:46pm

Collapse all instruments

#	Variable / Field Name	Field Label<br>*Field Note*	Field Attributes (Field Type, Validation, Choices, Calculations, etc.)
Instrument: **Summary Explanation of Research** (summary_explanation_of_research) Enabled as survey Collapse			
1	participant_id	Participant ID	text
2	consent	Do you want to complete the survey ?	yesno<br>1 Yes<br>0 No<br>Stop actions on 0
3	age_limit	Are you 18 years old or above?	yesno<br>1 Yes<br>0 No<br>Stop actions on 0
4	exclusion_eu	Are you from Europe or European Union (EU)?	yesno<br>1 Yes<br>0 No<br>Stop actions on 1
5	summary_explanation_of_research_complete	Section Header: *Form Status*<br>Complete?	dropdown<br>0 Incomplete<br>1 Unverified<br>2 Complete
Instrument: **Concern about COVID-19** (concern_about_covid19) Enabled as survey Collapse			
6	healthcare_worker	Are you a healthcare worker ?	yesno<br>1 Yes<br>0 No
7	family_member_covid	You or your family member had symptoms like COVID-19:	yesno<br>1 Yes<br>0 No<br>Custom alignment: LV
8	family_member_covid_test<br>Show the field ONLY if:<br>[family_member_covid] = '1'	You or your family member were tested for COVID-19 :	yesno<br>1 Yes<br>0 No<br>Custom alignment: LV
9	fam_mem_covid_dx<br>Show the field ONLY if:<br>[family_member_covid_test] = '1'	You or your family member were diagnosed with COVID-19 :	yesno<br>1 Yes<br>0 No<br>Custom alignment: LV

10	fm_mem_covid_hospitalized<br><br>Show the field ONLY if:<br>[fam_mem_covid_dx] = '1'	You or your family member were hospitalized with COVID-19 :	yesno<br>1 | Yes<br>0 | No<br><br>Custom alignment: LV
11	covid_follow	When did you start following COVID-19 actively ?	radio<br>1 | December 2019<br>2 | Mid-January 2020<br>3 | End of January 2020<br>4 | Mid-February 2020<br>5 | End of February 2020<br>6 | Mid-March 2020<br>7 | End of March 2020<br>8 | April 2020<br><br>Custom alignment: LH
12	covid_information_source	Which sources do you follow for COVID-19 updates ? (CHECK ALL THAT APPLY)	checkbox<br>1 | covid_information_source___1 | Television news channel<br>2 | covid_information_source___2 | Friends and family<br>3 | covid_information_source___3 | World health organization (WHO) website<br>4 | covid_information_source___4 | CDC (center for disease control) website<br>5 | covid_information_source___5 | Facebook<br>6 | covid_information_source___6 | WhatsApp<br>7 | covid_information_source___7 | City, state or other official website<br>8 | covid_information_source___8 | National health websites<br>9 | covid_information_source___9 | Your doctor or local hospital<br>10 | covid_information_source___10 | Scientific journal<br><br>Custom alignment: LV
13	confine	Section Header: *Stress Related to COVID-19 Pandemic*<br><br>Being confined at home with minimal social interaction is	radio (Matrix)<br>1 | Very stressful<br>2 | Stressful<br>3 | Sometimes stressful<br>4 | Mostly handled well<br>5 | Not stressful at all<br><br>Field Annotation: confine

14	job	Job situation or possibility of financial instability is	radio (Matrix)<br>1 Very stressful<br>2 Stressful<br>3 Sometimes stressful<br>4 Mostly handled well<br>5 Not stressful at all<br><br>Field Annotation: job
15	infection	The possibility of getting infected with COVID-19 is	radio (Matrix)<br>1 Very stressful<br>2 Stressful<br>3 Sometimes stressful<br>4 Mostly handled well<br>5 Not stressful at all<br><br>Field Annotation: infection
16	food	Possible shortage of food and other essentials in the coming months	radio (Matrix)<br>1 Very stressful<br>2 Stressful<br>3 Sometimes stressful<br>4 Mostly handled well<br>5 Not stressful at all<br><br>Field Annotation: food
17	death	One of your family members is at risk of serious complication due to COVID-19	radio (Matrix)<br>1 Very stressful<br>2 Stressful<br>3 Sometimes stressful<br>4 Mostly handled well<br>5 Not stressful at all<br><br>Field Annotation: death
18	hospital_access	Lack of access to a good medical facility	radio (Matrix)<br>1 Very stressful<br>2 Stressful<br>3 Sometimes stressful<br>4 Mostly handled well<br>5 Not stressful at all<br><br>Field Annotation: hospital_access
19	covid_state	Situation of COVID-19 at your state	radio (Matrix)<br>1 Very stressful<br>2 Stressful<br>3 Sometimes stressful<br>4 Mostly handled well<br>5 Not stressful at all<br><br>Field Annotation: COVID_state

20	sd	Section Header: *Prevention of Disease Spread*<br>Social distancing helps	radio (Matrix)<br>1 Strongly agree<br>2 Agree<br>3 Neutral<br>4 Disagree<br>5 Strongly disagree<br>Field Annotation: SD
21	ld	'Lockdown' or 'stay at home' orders are justified	radio (Matrix)<br>1 Strongly agree<br>2 Agree<br>3 Neutral<br>4 Disagree<br>5 Strongly disagree<br>Field Annotation: LD
22	hw	'Handwash for 20 seconds' whenever needed, is useful	radio (Matrix)<br>1 Strongly agree<br>2 Agree<br>3 Neutral<br>4 Disagree<br>5 Strongly disagree<br>Field Annotation: HW
23	fm	'Face mask' in public should be mandatory	radio (Matrix)<br>1 Strongly agree<br>2 Agree<br>3 Neutral<br>4 Disagree<br>5 Strongly disagree<br>Field Annotation: FM
24	er	One should go to Emergency/doctor immediately if he/she has symptoms like COVID-19	radio (Matrix)<br>1 Strongly agree<br>2 Agree<br>3 Neutral<br>4 Disagree<br>5 Strongly disagree<br>Field Annotation: ER
25	reopening	Reopening of your state is a right decision	radio (Matrix)<br>1 Strongly agree<br>2 Agree<br>3 Neutral<br>4 Disagree<br>5 Strongly disagree<br>Field Annotation: reopening

26	second_wave	Reopening would lead to a second spike of COVID-19 cases	radio (Matrix)<br>1 Strongly agree<br>2 Agree<br>3 Neutral<br>4 Disagree<br>5 Strongly disagree<br>Field Annotation: second spike
27	number	Section Header: *COVID cases*<br>Actual number of COVID-19 cases are much higher than the number of reported cases	radio (Matrix)<br>1 Strongly agree<br>2 Agree<br>3 Neutral<br>4 Disagree<br>5 Strongly disagree<br>Field Annotation: number
28	state	The situation of COVID-19 in your state is/was concerning	radio (Matrix)<br>1 Strongly agree<br>2 Agree<br>3 Neutral<br>4 Disagree<br>5 Strongly disagree<br>Field Annotation: state
29	belief	COVID-19 can be controlled by the end of 2020	radio (Matrix)<br>1 Strongly agree<br>2 Agree<br>3 Neutral<br>4 Disagree<br>5 Strongly disagree<br>Field Annotation: belief
30	concern_about_covid19_complete	Section Header: *Form Status*<br>Complete?	dropdown<br>0 Incomplete<br>1 Unverified<br>2 Complete
Instrument: **Knowledge On Covid19** (knowledge_on_covid19) Enabled as survey Collapse			
31	age	Section Header: *Who are at high risk for serious coronavirus infection ?*<br>Elderly people (age >60 years)	radio (Matrix)<br>1 Yes<br>0 No<br>Field Annotation: age
32	child	Children (0-5 years)	radio (Matrix)<br>1 Yes<br>0 No<br>Field Annotation: child
33	smoke	Smokers	radio (Matrix)<br>1 Yes<br>0 No<br>Field Annotation: smoke

34	dm	Diabetic patients	radio (Matrix)<br>1 Yes<br>0 No<br><br>Field Annotation: DM
35	immune	People without ability to fight infection	radio (Matrix)<br>1 Yes<br>0 No<br><br>Field Annotation: Immune
36	pregnant	Pregnant women	radio (Matrix)<br>1 Yes<br>0 No<br><br>Field Annotation: pregnant
37	hosp_rate	Section Header: *Knowledge on COVID-19*<br>Most COVID-19 patients need hospitalization	radio (Matrix)<br>1 Yes<br>0 No<br><br>Field Annotation: most
38	death_rate	One in five COVID-19 patients die	radio (Matrix)<br>1 Yes<br>0 No<br><br>Field Annotation: Die
39	incubation	It takes 2-3 weeks to have disease symptoms after getting coronavirus	radio (Matrix)<br>1 Yes<br>0 No<br><br>Field Annotation: incubation
40	corona	Before COVID-19, we did not know that coronavirus can affect human	radio (Matrix)<br>1 Yes<br>0 No<br><br>Field Annotation: corona
41	animal	We can get COVID-19 from animals	radio (Matrix)<br>1 Yes<br>0 No<br><br>Field Annotation: Animal
42	screening	Every person in the community should be tested for COVID-19	radio (Matrix)<br>1 Yes<br>0 No<br><br>Field Annotation: test
43	hand_wash	Section Header: *Which of the following helps to prevent COVID-19 ?*<br>Hand washing	radio (Matrix)<br>1 Yes<br>0 No<br><br>Field Annotation: prevent
44	climate	Hot and humid climate	radio (Matrix)<br>1 Yes<br>0 No<br><br>Field Annotation: climate

45	sunlight	Exposure to bright sunlight	radio (Matrix)<br>1 Yes<br>0 No<br><br>Field Annotation: sunlight
46	nasal_spray	Saline nasal spray	radio (Matrix)<br>1 Yes<br>0 No<br><br>Field Annotation: nasal spray
47	hot_beverages	Drinking hot beverages or alcohol	radio (Matrix)<br>1 Yes<br>0 No<br><br>Field Annotation: hot beverages
48	hand_sanitize	Hand sanitizer containing alcohol	radio (Matrix)<br>1 Yes<br>0 No<br><br>Field Annotation: Hand sanitize
49	baby_wipes	Baby wipes	radio (Matrix)<br>1 Yes<br>0 No<br><br>Field Annotation: Baby wipes
50	covid_symptoms	COVID-19 symptoms include (CHECK ALL THAT APPLY) :	checkbox<br>1 covid_symptoms___1 Fever<br>2 covid_symptoms___2 Cough<br>3 covid_symptoms___3 Shortness of breath<br>4 covid_symptoms___4 Body ache<br>5 covid_symptoms___5 Sore throat<br><br>Custom alignment: LV
51	vaccine_pcv_flu	Vaccines :	radio<br>1 Pneumonia vaccine offers some protection against COVID-19<br>2 Flu shot offers some protection against COVID-19<br>3 Both 1 and 2 are true<br>4 Both 1 and 2 are false<br><br>Custom alignment: LV
52	mask_gloves	Protective measures (CHECK ALL THAT APPLY) :	checkbox<br>1 mask_gloves___1 Face-mask prevents the spread of COVID-19<br>2 mask_gloves___2 Rubber gloves should be used while going outside (such as groceries)<br><br>Custom alignment: LV

53	social_distance_feet	Social distancing means keeping ____feet gap between two persons :	radio<br>1 3 feet<br>2 6 feet<br>3 10 feet<br>4 15 feet<br><br>Custom alignment: LV
54	isolation	If you had contact with a COVID-19 patient, you should be isolated for :	radio<br>1 One week<br>2 Two weeks<br>3 Three weeks<br>4 Four weeks<br><br>Custom alignment: LV
55	corona_live	Coronavirus can live on a surface for :	radio<br>1 Up to 6 hours<br>2 Few hours to 3 days<br>3 Up to 14 days<br><br>Custom alignment: LV
56	spread	COVID-19 usually spreads via (CHECK ALL THAT APPLY) :	checkbox<br>1 spread___1 Contact (by touch)<br>2 spread___2 Droplet (such as sneeze)<br>3 spread___3 Water (drinking water, shower)<br>4 spread___4 Wind/air<br><br>Custom alignment: LV
57	knowledge_on_covid19_complete	Section Header: *Form Status*<br>Complete?	dropdown<br>0 Incomplete<br>1 Unverified<br>2 Complete

Instrument: **Treatment of COVID-19** (treatment_of_covid19) Enabled as survey Collapse

58	chloroquin	Section Header: *Treatment*<br>Hydroxychloroquine (Plaquenil) is useful to treat COVID-19	radio (Matrix)<br>1 Strongly agree<br>2 Agree<br>3 Neutral<br>4 Disagree<br>5 Strongly disagree<br><br>Field Annotation: chloroquin
59	drug_trial	I am willing to participate in future drug trials for COVID-19	radio (Matrix)<br>1 Strongly agree<br>2 Agree<br>3 Neutral<br>4 Disagree<br>5 Strongly disagree<br><br>Field Annotation: drug trial

60	vaccine	I am willing to participate in future vaccine trials for COVID-19	radio (Matrix)<br>1 Strongly agree<br>2 Agree<br>3 Neutral<br>4 Disagree<br>5 Strongly disagree<br><br>Field Annotation: vaccine
61	hospital	Section Header: *Thoughts about your local hospital*<br>Your local hospital can treat very sick COVID-19 patients	radio (Matrix)<br>1 Strongly agree<br>2 Agree<br>3 Neutral<br>4 Disagree<br>5 Strongly disagree<br><br>Field Annotation: hospital
62	vent	Your local hospital has enough ventilators	radio (Matrix)<br>1 Strongly agree<br>2 Agree<br>3 Neutral<br>4 Disagree<br>5 Strongly disagree<br><br>Field Annotation: vent
63	administration	The government of your state/country acted promptly to prevent COVID-19	radio (Matrix)<br>1 Strongly agree<br>2 Agree<br>3 Neutral<br>4 Disagree<br>5 Strongly disagree<br><br>Field Annotation: administration
64	new_vaccine	Section Header: *Future hope*<br>How soon do you think that a vaccine against COVID-19 will be available?	radio (Matrix)<br>1 3 months<br>2 6 months<br>3 one year<br>4 Two years or more<br><br>Field Annotation: new vaccine
65	new_medication	How soon do you think that a medication to cure COVID-19 will be available?	radio (Matrix)<br>1 3 months<br>2 6 months<br>3 one year<br>4 Two years or more<br><br>Field Annotation: new_medication
66	covid_vaccine	Would you like to get COVID-19 vaccine, If available?	yesno<br>1 Yes<br>0 No

67	covid_vaccine_cause	What is your biggest concern on COVID-19 vaccine (even if you will take it)?	checkbox<br>1 covid_vaccine_cause___1 Possible side effects<br>2 covid_vaccine_cause___2 Doubt about the benefits or necessity<br>3 covid_vaccine_cause___3 Cost of the vaccine<br>4 covid_vaccine_cause___4 Not at risk for serious disease<br>5 covid_vaccine_cause___5 No specific cause/concern
68	flu_shot_y_n	Did you get a flu shot within last three years?	yesno<br>1 Yes<br>0 No<br><br>Custom alignment: LV
69	section_5_8	Since we now know how bad a viral infection can be, would you like to get Flu shot?	radio<br>1 Yes<br>0 No<br>2 Not sure<br><br>Custom alignment: LV
70	confidence_level	How confident you are about your answers: (0 is not confident at all and 10 is very confident)	radio<br>0 0<br>1 1<br>2 2<br>3 3<br>4 4<br>5 5<br>6 6<br>7 7<br>8 8<br>9 9<br>10 10<br><br>Custom alignment: LH
71	m_f	You are	radio<br>1 Male<br>2 Female<br>3 Prefer not to answer<br><br>Custom alignment: LV<br>Field Annotation: We would like to know a little about you (and those will not identify you)
72	your_age	Your age	radio<br>1 18-24 years<br>2 25-44 years<br>3 45-60 years<br>4 61-70 years<br>5 >70 years<br>6 Prefer not to answer<br><br>Custom alignment: LV

73	your_education	Your highest level of education	radio<br>1 Middle school or less<br>2 High School<br>3 Under graduate<br>4 Graduate<br>5 Masters<br>6 Doctorate<br>7 Professional degree (MD, MBA, LAWYER etc..)<br>8 Other<br>9 Prefer not to answer<br><br>Custom alignment: LV
74	your_race	You would like to identify yourself as	radio<br>1 Black<br>2 Hispanic/Latino<br>3 White<br>4 Asian (Chinese, Japanese, Korean, Filipino)<br>5 Asian Indian<br>6 Belongs to two or more races<br>7 Middle Eastern<br>8 Others<br>9 Prefer not to answer<br><br>Custom alignment: LV
75	financial_status	You consider the financial status of your family as	radio<br>1 Lower middle class/ poor<br>2 Middle class<br>3 Upper middle class<br>4 Wealthy<br><br>Custom alignment: LV
76	country	Do you live in the USA?	yesno<br>1 Yes<br>0 No
77	country_not_usa<br><br>Show the field ONLY if:<br>[country] = '0'	Name of your country	text
78	state_usa<br><br>Show the field ONLY if:<br>[country] = '1'	Name of your State	text
79	treatment_of_covid19_complete	Section Header: *Form Status*<br>Complete?	dropdown<br>0 Incomplete<br>1 Unverified<br>2 Complete
