# Supplementary material for: Perceived Stress, Knowledge, and Preventive Behaviors in Indian versus US-based Participants During COVID-19: A Survey Study
Source: Front Public Health. 2021 Sep 13;9:687864. doi: 10.3389/fpubh.2021.687864 (PMC8473728; doi:10.3389/fpubh.2021.687864)
Supplement: Supplementary file 2 [file Data_Sheet_2.PDF]

10	fm_mem_covid_hospitalized<br>Show the field ONLY if:<br>[fam_mem_covid_dx] = '1'	You or your family member were hospitalized with COVID-19 :	yesno<br>1 Yes<br>0 No<br>Custom alignment: LV
11	covid_follow	When did you start following COVID-19 actively ?	radio<br>1 December 2019<br>2 Mid-January 2020<br>3 End of January 2020<br>4 Mid-February 2020<br>5 End of February 2020<br>6 Mid-March 2020<br>7 End of March 2020<br>8 April 2020<br>Custom alignment: LH
12	covid_information_source	Which sources do you follow for COVID-19 updates ? (CHECK ALL THAT APPLY)	checkbox<br>1 covid_information_source___1 Television news channel<br>2 covid_information_source___2 Friends and family<br>3 covid_information_source___3 World health organization (WHO) website<br>4 covid_information_source___4 CDC (center for disease control) website<br>5 covid_information_source___5 Facebook<br>6 covid_information_source___6 WhatsApp<br>7 covid_information_source___7 City, state or other official website<br>8 covid_information_source___8 National health websites<br>9 covid_information_source___9 Your doctor or local hospital<br>10 covid_information_source___10 Scientific journal<br>Custom alignment: LV
13	confine	Section Header: *Stress Related to COVID-19 Pandemic*<br>Being confined at home with minimal social interaction is	radio (Matrix)<br>1 Very stressful<br>2 Stressful<br>3 Sometimes stressful<br>4 Mostly handled well<br>5 Not stressful at all<br>Field Annotation: confine

14	job	Job situation or possibility of financial instability is	radio (Matrix)<br>1 Very stressful<br>2 Stressful<br>3 Sometimes stressful<br>4 Mostly handled well<br>5 Not stressful at all<br><br>Field Annotation: job
15	infection	The possibility of getting infected with COVID-19 is	radio (Matrix)<br>1 Very stressful<br>2 Stressful<br>3 Sometimes stressful<br>4 Mostly handled well<br>5 Not stressful at all<br><br>Field Annotation: infection
16	food	Possible shortage of food and other essentials in the coming months	radio (Matrix)<br>1 Very stressful<br>2 Stressful<br>3 Sometimes stressful<br>4 Mostly handled well<br>5 Not stressful at all<br><br>Field Annotation: food
17	death	One of your family members is at risk of serious complication due to COVID-19	radio (Matrix)<br>1 Very stressful<br>2 Stressful<br>3 Sometimes stressful<br>4 Mostly handled well<br>5 Not stressful at all<br><br>Field Annotation: death
18	hospital_access	Lack of access to a good medical facility	radio (Matrix)<br>1 Very stressful<br>2 Stressful<br>3 Sometimes stressful<br>4 Mostly handled well<br>5 Not stressful at all<br><br>Field Annotation: hospital_access
19	covid_state	Situation of COVID-19 at your state	radio (Matrix)<br>1 Very stressful<br>2 Stressful<br>3 Sometimes stressful<br>4 Mostly handled well<br>5 Not stressful at all<br><br>Field Annotation: COVID_state
