# Supplementary material for: Perceived Stress, Knowledge, and Preventive Behaviors in Indian versus US-based Participants During COVID-19: A Survey Study
Source: Front Public Health. 2021 Sep 13;9:687864. doi: 10.3389/fpubh.2021.687864 (PMC8473728; doi:10.3389/fpubh.2021.687864)
Supplement: Supplementary file 3 [file Data_Sheet_3.PDF]

26	second_wave	Reopening would lead to a second spike of COVID-19 cases	radio (Matrix)<br>1 Strongly agree<br>2 Agree<br>3 Neutral<br>4 Disagree<br>5 Strongly disagree<br><br>Field Annotation: second spike
27	number	Section Header: *COVID cases*<br>Actual number of COVID-19 cases are much higher than the number of reported cases	radio (Matrix)<br>1 Strongly agree<br>2 Agree<br>3 Neutral<br>4 Disagree<br>5 Strongly disagree<br><br>Field Annotation: number
28	state	The situation of COVID-19 in your state is/was concerning	radio (Matrix)<br>1 Strongly agree<br>2 Agree<br>3 Neutral<br>4 Disagree<br>5 Strongly disagree<br><br>Field Annotation: state
29	belief	COVID-19 can be controlled by the end of 2020	radio (Matrix)<br>1 Strongly agree<br>2 Agree<br>3 Neutral<br>4 Disagree<br>5 Strongly disagree<br><br>Field Annotation: belief
30	concern_about_covid19_complete	Section Header: *Form Status*<br>Complete?	dropdown<br>0 Incomplete<br>1 Unverified<br>2 Complete
Instrument: **Knowledge On Covid19** (knowledge_on_covid19) Enabled as survey Collapse			
31	age	Section Header: *Who are at high risk for serious coronavirus infection ?*<br>Elderly people (age >60 years)	radio (Matrix)<br>1 Yes<br>0 No<br><br>Field Annotation: age
32	child	Children (0-5 years)	radio (Matrix)<br>1 Yes<br>0 No<br><br>Field Annotation: child
33	smoke	Smokers	radio (Matrix)<br>1 Yes<br>0 No<br><br>Field Annotation: smoke

34	dm	Diabetic patients	radio (Matrix)<br>1 Yes<br>0 No<br><br>Field Annotation: DM
35	immune	People without ability to fight infection	radio (Matrix)<br>1 Yes<br>0 No<br><br>Field Annotation: Immune
36	pregnant	Pregnant women	radio (Matrix)<br>1 Yes<br>0 No<br><br>Field Annotation: pregnant
37	hosp_rate	Section Header: *Knowledge on COVID-19*<br>Most COVID-19 patients need hospitalization	radio (Matrix)<br>1 Yes<br>0 No<br><br>Field Annotation: most
38	death_rate	One in five COVID-19 patients die	radio (Matrix)<br>1 Yes<br>0 No<br><br>Field Annotation: Die
39	incubation	It takes 2-3 weeks to have disease symptoms after getting coronavirus	radio (Matrix)<br>1 Yes<br>0 No<br><br>Field Annotation: incubation
40	corona	Before COVID-19, we did not know that coronavirus can affect human	radio (Matrix)<br>1 Yes<br>0 No<br><br>Field Annotation: corona
41	animal	We can get COVID-19 from animals	radio (Matrix)<br>1 Yes<br>0 No<br><br>Field Annotation: Animal
42	screening	Every person in the community should be tested for COVID-19	radio (Matrix)<br>1 Yes<br>0 No<br><br>Field Annotation: test
43	hand_wash	Section Header: *Which of the following helps to prevent COVID-19 ?*<br>Hand washing	radio (Matrix)<br>1 Yes<br>0 No<br><br>Field Annotation: prevent
44	climate	Hot and humid climate	radio (Matrix)<br>1 Yes<br>0 No<br><br>Field Annotation: climate

45	sunlight	Exposure to bright sunlight	radio (Matrix)<br>1 Yes<br>0 No<br><br>Field Annotation: sunlight
46	nasal_spray	Saline nasal spray	radio (Matrix)<br>1 Yes<br>0 No<br><br>Field Annotation: nasal spray
47	hot_beverages	Drinking hot beverages or alcohol	radio (Matrix)<br>1 Yes<br>0 No<br><br>Field Annotation: hot beverages
48	hand_sanitize	Hand sanitizer containing alcohol	radio (Matrix)<br>1 Yes<br>0 No<br><br>Field Annotation: Hand sanitize
49	baby_wipes	Baby wipes	radio (Matrix)<br>1 Yes<br>0 No<br><br>Field Annotation: Baby wipes
50	covid_symptoms	COVID-19 symptoms include (CHECK ALL THAT APPLY) :	checkbox<br>1 covid_symptoms___1 Fever<br>2 covid_symptoms___2 Cough<br>3 covid_symptoms___3 Shortness of breath<br>4 covid_symptoms___4 Body ache<br>5 covid_symptoms___5 Sore throat<br><br>Custom alignment: LV
51	vaccine_pcv_flu	Vaccines :	radio<br>1 Pneumonia vaccine offers some protection against COVID-19<br>2 Flu shot offers some protection against COVID-19<br>3 Both 1 and 2 are true<br>4 Both 1 and 2 are false<br><br>Custom alignment: LV
52	mask_gloves	Protective measures (CHECK ALL THAT APPLY) :	checkbox<br>1 mask_gloves___1 Face-mask prevents the spread of COVID-19<br>2 mask_gloves___2 Rubber gloves should be used while going outside (such as groceries)<br><br>Custom alignment: LV

53	social_distance_feet	Social distancing means keeping ____feet gap between two persons :	radio<br>1 3 feet<br>2 6 feet<br>3 10 feet<br>4 15 feet<br>Custom alignment: LV
54	isolation	If you had contact with a COVID-19 patient, you should be isolated for :	radio<br>1 One week<br>2 Two weeks<br>3 Three weeks<br>4 Four weeks<br>Custom alignment: LV
55	corona_live	Coronavirus can live on a surface for :	radio<br>1 Up to 6 hours<br>2 Few hours to 3 days<br>3 Up to 14 days<br>Custom alignment: LV
56	spread	COVID-19 usually spreads via (CHECK ALL THAT APPLY) :	checkbox<br>1 spread___1 Contact (by touch)<br>2 spread___2 Droplet (such as sneeze)<br>3 spread___3 Water (drinking water, shower)<br>4 spread___4 Wind/air<br>Custom alignment: LV
57	knowledge_on_covid19_complete	Section Header: *Form Status*<br>Complete?	dropdown<br>0 Incomplete<br>1 Unverified<br>2 Complete
Instrument: **Treatment of COVID-19** (treatment_of_covid19) Enabled as survey			Collapse
58	chloroquin	Section Header: *Treatment*<br>Hydroxychloroquine (Plaquenil) is useful to treat COVID-19	radio (Matrix)<br>1 Strongly agree<br>2 Agree<br>3 Neutral<br>4 Disagree<br>5 Strongly disagree<br>Field Annotation: chloroquin
59	drug_trial	I am willing to participate in future drug trials for COVID-19	radio (Matrix)<br>1 Strongly agree<br>2 Agree<br>3 Neutral<br>4 Disagree<br>5 Strongly disagree<br>Field Annotation: drug trial
