# Supplementary material for: Perceived Stress, Knowledge, and Preventive Behaviors in Indian versus US-based Participants During COVID-19: A Survey Study
Source: Front Public Health. 2021 Sep 13;9:687864. doi: 10.3389/fpubh.2021.687864 (PMC8473728; doi:10.3389/fpubh.2021.687864)
Supplement: Supplementary file 4 [file Data_Sheet_4.PDF]

20	sd	Section Header: *Prevention of Disease Spread*<br>Social distancing helps	radio (Matrix)<br>1 Strongly agree<br>2 Agree<br>3 Neutral<br>4 Disagree<br>5 Strongly disagree<br><br>Field Annotation: SD
21	ld	'Lockdown' or 'stay at home' orders are justified	radio (Matrix)<br>1 Strongly agree<br>2 Agree<br>3 Neutral<br>4 Disagree<br>5 Strongly disagree<br><br>Field Annotation: LD
22	hw	'Handwash for 20 seconds' whenever needed, is useful	radio (Matrix)<br>1 Strongly agree<br>2 Agree<br>3 Neutral<br>4 Disagree<br>5 Strongly disagree<br><br>Field Annotation: HW
23	fm	'Face mask' in public should be mandatory	radio (Matrix)<br>1 Strongly agree<br>2 Agree<br>3 Neutral<br>4 Disagree<br>5 Strongly disagree<br><br>Field Annotation: FM
24	er	One should go to Emergency/doctor immediately if he/she has symptoms like COVID-19	radio (Matrix)<br>1 Strongly agree<br>2 Agree<br>3 Neutral<br>4 Disagree<br>5 Strongly disagree<br><br>Field Annotation: ER
25	reopening	Reopening of your state is a right decision	radio (Matrix)<br>1 Strongly agree<br>2 Agree<br>3 Neutral<br>4 Disagree<br>5 Strongly disagree<br><br>Field Annotation: reopening
