# Supplementary material for: Perceived Stress, Knowledge, and Preventive Behaviors in Indian versus US-based Participants During COVID-19: A Survey Study
Source: Front Public Health. 2021 Sep 13;9:687864. doi: 10.3389/fpubh.2021.687864 (PMC8473728; doi:10.3389/fpubh.2021.687864)
Supplement: Supplementary file 5 [file Data_Sheet_5.PDF]

COVID-related perceptions	India			USA			Power of the study
	N	Mean	SD	N	Mean	SD	
Stress	242	7.0140	1.51259	530	6.0785	1.61921	1.00
Knowledge	242	5.1853	1.94922	530	7.8201	1.34934	1.00
Preventive behavior	242	8.8452	1.30489	530	8.3412	2.09073	0.99

Supplemental Table 1: The power of our study was estimated (non-directional two-sided analyses) for each of the COVID-perception metrics, based independent sample t-test that compared scores between the IND-P and US-P, which had unequal sample size. With an alpha = .05, the projected power of the study above .8 was considered statistically significant.
