# Supplementary material for: Perceived Stress, Knowledge, and Preventive Behaviors in Indian versus US-based Participants During COVID-19: A Survey Study
Source: Front Public Health. 2021 Sep 13;9:687864. doi: 10.3389/fpubh.2021.687864 (PMC8473728; doi:10.3389/fpubh.2021.687864)
Supplement: Supplementary file 6 [file Data_Sheet_6.PDF]

STROBE Statement—Checklist of items that should be included in reports of ***cross-sectional studies***

	Item No	Recommendation
**Title and abstract**	1	(*a*) Indicate the study's design with a commonly used term in the title or the abstract<br>We described the type of study in the title.
		(*b*) Provide in the abstract an informative and balanced summary of what was done and what was found<br>We summarized the major findings in the abstract.
**Introduction**		
Background/rationale	2	Explain the scientific background and rationale for the investigation being reported<br>The background and rationale were based on an updated literature review.
Objectives	3	State specific objectives, including any prespecified hypotheses<br>The aims and hypotheses were described in the last paragraphs of the introduction.
**Methods**		
Study design	4	Present key elements of study design early in the paper<br>We explained.
Setting	5	Describe the setting, locations, and relevant dates, including periods of recruitment, exposure, follow-up, and data collection<br>We conducted an online survey between the end of May 2020 to October 2020.
Participants	6	(*a*) Give the eligibility criteria, and the sources and methods of selection of participants<br>The participants must be from India or the USA and above 18 years of age. It was an anonymous public survey.
Variables	7	Clearly define all outcomes, exposures, predictors, potential confounders, and effect modifiers. Give diagnostic criteria, if applicable.<br>The primary outcome of the study was the difference in knowledge, stress, preventive behaviors, and perceived threat between participants from India and the USA.
Data sources/ measurement	8*	For each variable of interest, give sources of data and details of methods of assessment (measurement). Describe comparability of assessment methods if there is more than one group<br>Our data source was the survey responses collected in Redcap server. Most of the responses were either in yes vs. no dichotomous variables or on a 1-5 Likert scale. We used t-tests to compare continuous variables such as COVID perception scores, and the Kruskal Wallis test was used to compare the ordinal dependent variables.
Bias	9	Describe any efforts to address potential sources of bias<br>Selection bias was a concern. To address that, we made considerable efforts to reach out to people across India and the

		USA. Researchmatch, a research portal, was primarily used to recruit US-P. We randomly sent a request to participate in the Redcap survey to a pool of 150000 volunteers of all 50 US states. However, a similar web-based tool was not available that could be used to recruit IND-P. Thus, we mostly relied upon social media like Facebook. We distributed the survey in Facebook groups representing 44 different cities and spread out at different parts of India. Although it was an anonymous survey, we received feedback from the participants several times after completion of the study, which helped us recognize the diverse geographic distribution of the participants across India.
Study size	10	Explain how the study size was arrived at<br>Considering an unequal sample size between India and the USA, power was estimated for each of the COVID-perception metrics (stress, knowledge, and preventive behavior), based on a two-sided independent sample t-test that compared COVID-perception scores between the IND-P and US-P. With an alpha = .05, each of the three comparative analyses' projected power was above the statistical significance cut-off .8 (Supplemental Table 1).
Quantitative variables	11	Explain how quantitative variables were handled in the analyses. If applicable, describe which groupings were chosen and why<br>We described statistical methods in detail, including the factor analyses, that we used to abbreviate the metrics on stress, knowledge, preventive behavior into three normalized nominal scores (0-10), respectively. The study participants were grouped according to their age, gender, education, and financial status. A detailed description is available at Mendeley data repository.
Statistical methods	12	(*a*) Describe all statistical methods, including those used to control for confounding<br>We described statistical methods in detail, including the factor analyses, that we used to consolidate stress, knowledge, and preventive-behaviors and perceived threat metrics into single normalized continuous variables, respectively. We also described the rationale of using Kruskal–Wallis (KW) test and generalized linear regression (GLM). GLM was controlled for confounding factors like age, gender, family income and education level of the participants.
		(*b*) Describe any methods used to examine subgroups and interactions<br>Categories among socio-economic factors like age, education level, family income were distributed on an ordinal scale. Kruskal–Wallis test is used, (non-parametric counterpart of ANOVA) which considers subgroup interactions.
		(*c*) Explain how missing data were addressed<br>KW tests and GLM listwise excluded the missing value, while for frequency calculations, missing values were excluded, and the analyses were based on the remaining data.

		(*d*) If applicable, describe analytical methods taking account of sampling strategy<br>Not applicable.
		(*e*) Describe any sensitivity analyses<br>Not applicable.
**Results**		
Participants	13*	(a) Report numbers of individuals at each stage of study—eg numbers potentially eligible, examined for eligibility, confirmed eligible, included in the study, completing follow-up, and analysed
		(b) Give reasons for non-participation at each stage<br>**13 (a) (b)** In the first few lines of the result section, we explained.
		(c) Consider use of a flow diagram<br>We will be happy to add if the reviewers/editors recommend it. Since it was just one step, we decided to describe it in words.
Descriptive data	14*	(a) Give characteristics of study participants (eg demographic, clinical, social) and information on exposures and potential confounders<br>We described in the result section and displayed in Table 1.
		(b) Indicate number of participants with missing data for each variable of interest<br>Table 1 displayed N for each of the socio-demographic predictors, and the difference between eligible study participants (N=773) should be considered as the missing value.
Outcome data	15*	Report numbers of outcome events or summary measures<br>We described the difference in stress, knowledge, and preventive behavior between Indian and US-based participants in the methods and result section.
Main results	16	(*a*) Give unadjusted estimates and, if applicable, confounder-adjusted estimates and their precision (eg, 95% confidence interval). Make clear which confounders were adjusted for and why they were included<br>Our study analyses were based on T-tests, Kruskal Wallis test and generalized linear regression.
		(*b*) Report category boundaries when continuous variables were categorized<br>We computed four continuous variables for stress, knowledge, preventive behaviors, and perceived threat. All the variables were in the 0-10 range. We explained that in methods.
		(*c*) If relevant, consider translating estimates of relative risk into absolute risk for a meaningful time period<br>Not applicable.
Other analyses	17	Report other analyses done—eg analyses of subgroups and interactions, and sensitivity analyses<br>Not applicable.

**Discussion**		
Key results	18	Summarise key results with reference to study objectives<br>Please review the last paragraph of the discussion.
Limitations	19	Discuss limitations of the study, taking into account sources of potential bias or imprecision. Discuss both direction and magnitude of any potential bias.<br>One of the limitations of this study is generalizability. Considering the population of India and the USA, it cannot be said that these survey results represent the general perception of average Indians or Americans. Instead, this study report should be considered as a trend and needs to be externally validated with a large sample study to accept the results as a general trend.
Interpretation	20	Give a cautious overall interpretation of results considering objectives, limitations, multiplicity of analyses, results from similar studies, and other relevant evidence<br>In the last paragraph of the discussion, we summarized an overall interpretation.
Generalisability	21	Discuss the generalisability (external validity) of the study results<br>We performed cross-validation.
**Other information**		
Funding	22	Give the source of funding and the role of the funders for the present study and, if applicable, for the original study on which the present article is based.<br>Not applicable.

*Give information separately for exposed and unexposed groups.

Note: An Explanation and Elaboration article discusses each checklist item and gives methodological background and published examples of transparent reporting. The STROBE checklist is best used in conjunction with this article (freely available on the Web sites of PLoS Medicine at http://www.plosmedicine.org/, Annals of Internal Medicine at http://www.annals.org/, and Epidemiology at http://www.epidem.com/). Information on the STROBE Initiative is available at www.strobe-statement.org.
